# Mediation Analysis of Inflammatory Biomarkers in the Association Between Metabolic Dysfunction–Associated Steatotic Liver Disease and Sarcopenia Among US Adults With Type 2 Diabetes or Prediabetes

**DOI:** 10.1155/ije/3157645

**Published:** 2026-11-15

**Authors:** Lu Long, Zhiwen Shu, Weixue Wang, Lei He, Yuwen Jiang, Qing Li, Xiaozhong Li, Juxiang Li

**Affiliations:** ^1^ Department of Cardiovascular Medicine The Second Affiliated Hospital Jiangxi Medical College Nanchang University Nanchang Jiangxi China ncu.edu.cn; ^2^ Department of Orthopedics The Second Affiliated Hospital Jiangxi Medical College Nanchang University Nanchang Jiangxi China ncu.edu.cn

**Keywords:** MASLD, NHANES, prediabetes, sarcopenia, type 2 diabetes mellitus

## Abstract

**Background:**

Metabolic dysfunction–associated steatotic liver disease (MASLD) is strongly linked to metabolic disorders, including insulin resistance and obesity, yet its association with sarcopenia, particularly in individuals with type 2 diabetes (T2DM) or prediabetes, remains poorly characterized. This study examined the association between MASLD and sarcopenia in U.S. adults with T2DM or prediabetes and investigated the potential mediating role of inflammatory biomarkers.

**Methods:**

Data were obtained from the National Health and Nutrition Examination Survey (NHANES) 1999–2006 and 2011–2018 cycles. Multivariable logistic regression and restricted cubic spline analyses were conducted to investigate the association between US FLI and sarcopenia, while mediation analysis was performed to explore the potential mediating role of inflammatory biomarkers in this association.

**Results:**

Among the 2093 patients (weighted 9,501,956) with T2DM or prediabetes, 452 (17.10%) had sarcopenia. MASLD remained significantly associated with sarcopenia (OR = 2.12; 95% CI: 1.56–2.86) after fully adjusting for potential confounders. Restricted cubic spline analysis showed no evidence of a nonlinear association between US FLI and sarcopenia (*p* for nonlinearity = 0.81). Mediation analyses yielded estimated mediation proportions of 10.35%, 9.14%, 8.98%, 6.47%, 4.28%, and 2.54% for the red cell distribution width‐to‐albumin ratio, white blood cell count, serum albumin, neutrophil percentage‐to‐albumin ratio, red blood cell distribution width, and systemic inflammation response index, respectively, in the association between MASLD and sarcopenia.

**Conclusion:**

MASLD was significantly associated with sarcopenia among individuals with T2DM or prediabetes. Mediation analyses suggested a potential involvement of inflammatory biomarkers in this association.

## 1. Introduction

Sarcopenia refers to age‐related declines in muscle mass, muscle strength, and function, which are important clinical symptoms. The European Working Group on Sarcopenia in older people defines it as “a syndrome characterized by progressive and generalized loss of skeletal muscle mass and strength, with a risk of adverse outcomes such as physical disability, poor quality of life, and death” [1]. The reported prevalence of sarcopenia ranges from 5% to 13% among individuals aged 60–70 years and from 11% to 50% among those aged > 80 years [2]. Metabolic dysfunction–associated steatotic liver disease (MASLD), formerly known as nonalcoholic fatty liver disease (NAFLD), is the most prevalent chronic liver disease worldwide and is estimated to affect approximately 32% of the global adult population [3]. The association between MASLD and sarcopenia has garnered significant research interest in recent years. Previous studies have demonstrated that sarcopenia is associated with an increased prevalence of MASLD and more severe MASLD‐related fibrosis, independent of established risk factors [4, 5].

Chronic inflammation is widely recognized as a potential link between various metabolic abnormalities. In MASLD, steatotic hepatocytes and hepatic immune cells can release proinflammatory factors such as tumor necrosis factor‐α (TNF‐α) and interleukin‐6 (IL‐6), potentially contributing to a systemic, low‐grade inflammatory state [6, 7]. These inflammatory mediators may contribute to impaired muscle protein synthesis and muscle dysfunction [8, 9]. Previous cross‐sectional studies have reported significantly higher levels of circulating inflammatory markers among individuals with NAFLD or sarcopenia [10, 11]. Consistently, Hong et al. reported that individuals with sarcopenia exhibited higher levels of high‐sensitivity C‐reactive protein [12]. Nevertheless, whether inflammation plays a mediating role in the association between MASLD and sarcopenia remains unclear, particularly in large population‐based studies. According to the International Diabetes Federation (IDF), an estimated 537 million people were living with diabetes globally in 2021 [13]. Diabetes is characterized by insulin resistance and/or inadequate insulin secretion, resulting in persistent hyperglycemia [14, 15]. Notably, emerging evidence indicates that insulin resistance may represent a shared pathophysiological mechanism underlying both MASLD and sarcopenia [16, 17]. Correspondingly, previous studies have reported a higher prevalence of both sarcopenia and MASLD among individuals with diabetes [18, 19].

Therefore, this study aimed to investigate the potential mediating role of chronic inflammation in the association between MASLD and sarcopenia among U.S. adults with type 2 diabetes (T2DM) or prediabetes. The specific objectives were (1) to assess the association between MASLD and sarcopenia and (2) to examine the potential mediating role of inflammatory biomarkers in this association.

## 2. Materials and Methods

### 2.1. Data Source and Population

Data from the National Health and Nutrition Examination Survey (NHANES), a program designed to assess the nutritional status and general health of the U.S. population, were used in this cross‐sectional study. Every two years, the survey uses a sophisticated multistage probability sampling technique to gather data from noninstitutionalized civilians in the U.S. We used data from eight NHANES cycles (1999–2006 and 2011–2018) involving adults in the United States. Of the 80,630 individuals in these eight cycles, 11,632 had either prediabetes or T2DM. Participants were excluded according to the following criteria: (1) age < 20 years, (2) participants with missing data on diagnostic components of MASLD and sarcopenia, (3) those who were heavy alcohol consumers or tested positive for hepatitis B surface antigen, hepatitis C antibody, or hepatitis C RNA, and (4) pregnant participants. After removing the aforementioned participants, a total of 2093 participants were included in this cross‐sectional study (Figure 1).

**FIGURE 1:**
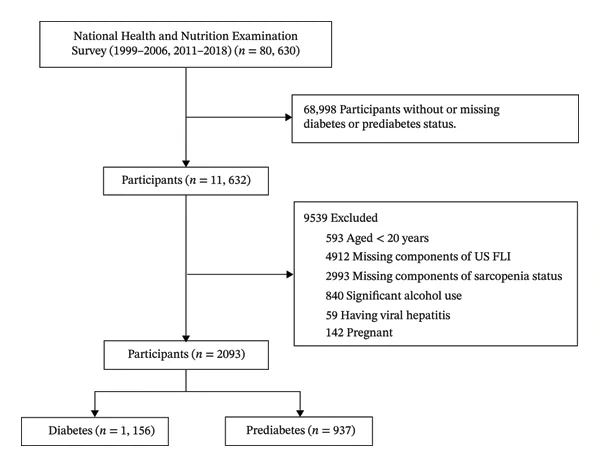
Flowchart of study participants.

### 2.2. Definition of T2DM or Prediabetes

The American Diabetes Association defines T2DM as a self‐reported diagnosis, use of insulin or oral hypoglycemic medicine, fasting blood glucose (FBG) ≥ 126 mg/dL, or HbA1c ≥ 6.5%. Prediabetes is diagnosed by self‐reported prediabetes status or having FBG between 100 mg/dL and 125 mg/dL, or HbA1c between 5.7% and 6.4% [20].

### 2.3. Definition of MASLD

Hepatic steatosis was assessed using the U.S. Fatty Liver Index (US FLI), a validated noninvasive predictive model. The specific definition of the US FLI is as follows: US FLI = (*e* ^*a*^)/(1 + *e* ^*a*^) × 100, wherein *a*  =   −0.8073   ×  non‐Hispanic black  +  0.3458 × Mexican American  +  0.0093  ×  age  +  0.6151  ×  loge (GGT) +0.0249 × waist circumference + 1.1792 × loge (insulin) + 0.8242 × loge (glucose) − 14.7812.

The predictive performance of this model was confirmed in previous studies, with an area under the receiver operating characteristic curve of 0.80 (95% confidence interval [CI], 0.77–0.83) and the cutoff value set at ≥ 30 for identifying patients with hepatic steatosis [21]. MASLD was defined as the presence of hepatic steatosis (US FLI ≥ 30) together with at least one cardiometabolic risk factor. These risk factors include overweight/obesity, insulin resistance, hypertension, dyslipidemia, and/or low high‐density lipoprotein cholesterol levels. Participants with other identifiable causes of chronic liver disease were excluded, including viral hepatitis (positive hepatitis B surface antigen, hepatitis C antibody, or hepatitis C RNA) and heavy alcohol consumption (≥ 3 drinks/day for women or ≥ 4 drinks/day for men) or binge drinking (≥ 5 drinks on a single occasion) [22].

### 2.4. Definition of Sarcopenia

Dual‐energy X‐ray absorptiometry (DXA) is considered a standard method for assessing body composition. Based on the data obtained from DXA measurements, the lean tissue weight of both arms and lower limbs is added together to determine the appendicular lean mass (ALM). This study employed the diagnostic criteria for sarcopenia established by the Foundation for the National Institutes of Health, which uses the ratio of ALM to BMI as a determinant (male < 0.789; female < 0.512) [23].

### 2.5. Definitions of Systemic Inflammatory Biomarkers

To comprehensively assess the systemic inflammatory status, the present study employed six inflammatory biomarkers: the red cell distribution width‐to‐albumin ratio (RAR), red cell distribution width (RDW), serum albumin, white blood cell count (WBC), neutrophil percentage‐to‐albumin ratio (NPAR), and systemic inflammation response index (SIRI). RAR was calculated as the ratio of RDW to serum albumin concentration [24], whereas NPAR was derived from neutrophil percentage and albumin level [25]. Both RAR and NPAR are recognized as composite indicators reflecting inflammatory activity and nutritional status [26, 27]. SIRI was applied to evaluate systemic inflammation based on neutrophil, lymphocyte, and monocyte counts [28]. The relevant detailed calculation methods are as follows:

 RAR = (RDW(%))/(albumin(g/L))
 NPAR = (neutrophil percentage(%))/(albumin(g/L))
 SIRI = (neutrophil counts∗monocyte counts)/(lymphocyte counts)

### 2.6. Other Definitions

Demographic characteristics, including age, sex, race/ethnicity, poverty‐to‐income ratio (PIR) (< 1.0, 1.0–3.0, and > 3.0) [29], and educational attainment, were obtained from NHANES data. Smoking status was categorized into never smokers (defined as < 100 cigarettes in a lifetime), current smokers, and former smokers (defined as having smoked ≥ 100 cigarettes in their lifetime and having quit smoking). Physical activity levels were classified as active or inactive. Hyperlipidemia (HL) was defined as meeting any of the following criteria: a total cholesterol level of 200 mg/dL or above, a low‐density lipoprotein cholesterol level of 130 mg/dL or higher, triglycerides (TG) of 150 mg/dL or more, a high‐density lipoprotein cholesterol level below 40 mg/dL for males and 50 mg/dL for women, or a previous diagnosis of HL [30]. Hypertension was defined as an average systolic blood pressure ≥ 130 mmHg or diastolic blood pressure ≥ 80 mmHg based on three measurements, or a prior diagnosis of hypertension [31].

### 2.7. Sensitivity Analysis

To evaluate the robustness of our findings, sensitivity analyses were performed by redefining MASLD using the Hepatic Steatosis Index (HSI), the Zhejiang University Index (ZJU), and the Framingham Steatosis Index (FSI) as alternative surrogate indices of hepatic steatosis. The HSI, introduced by Lee et al. in Korea in 2010, incorporates the ALT/AST ratio, BMI, sex, and diabetes mellitus status as key components [32]. The ZJU index was crafted in China and encompasses metrics like BMI, FBG, and TG, along with the ratio of ALT/AST [33]. Furthermore, the FSI was developed by Long and colleagues in 2016 using CT‐detected hepatic steatosis as the reference outcome [34]. Detailed calculation formulas for the HSI, ZJU, and FSI are provided in Table S1. Prior research indicated that individuals were considered to have hepatic steatosis when their HSI score was > 36 [32], ZJU score was > 38 [33], or FSI score was > −1.068 [34, 35], provided that there were no other known causes of chronic liver disease, and that they did not engage in heavy alcohol consumption.

### 2.8. Statistical Analysis

In this study, eight NHANES study cycles were used, and appropriate sampling weights and adjustment factors were applied according to the NHANES analysis and reporting guidelines. Categorical variables were presented as unweighted frequencies (weighted percentages), while all continuous variables were expressed as medians with interquartile ranges (IQRs), given their nonnormal distributions. To assess the relationships between MASLD and sarcopenia, multivariable logistic regression models were used, generating odds ratios (ORs) and 95% CIs. Potential confounders were selected a priori based on previous literature and clinical relevance [36, 37]. The crude model did not account for any covariates. Model 1 accounted for basic demographic variables (age, sex, and race), whereas Model 2 further incorporated socioeconomic, lifestyle‐related, and health‐related covariates, including educational level, PIR, smoking status, physical activity, hypertension, and dyslipidemia. Furthermore, the nonlinear relationships between MASLD and sarcopenia risk were assessed with restricted cubic spline (RCS) analysis.

Weighted multivariable linear regression models were used to evaluate the associations between MASLD status and continuous inflammatory biomarkers, whereas weighted multivariable logistic regression models were applied to examine the associations between inflammatory biomarkers and sarcopenia. To enhance the interpretability of ORs and regression coefficients (β), the original RAR values were rescaled by dividing them by 0.1. This rescaling procedure was implemented to address the issue of extreme OR estimates that might arise from the physiologically constrained range of RAR. We also utilized the “mediation” package in R to quantitatively assess the potential mediating effects of six biomarkers—RAR, RDW, serum albumin, WBC, NPAR, and SIRI—on the association between MASLD and sarcopenia.

Stratified analyses were performed according to sex, age (< 60 or ≥ 60 years), PIR, smoking status, physical activity, and hypertension (yes or no). R (Version 4.4.2) was utilized for statistical analysis, with a two‐sided significance threshold of *p* < 0.05.

## 3. Results

### 3.1. Baseline Characteristics

The characteristics of the study participants are summarized in Table 1. A total of 2093 adults with T2DM or prediabetes were included, comprising 1259 participants with MASLD and 834 without MASLD. The median age of the participants was 51.0 years (IQR, 43.0–58.0), and the median BMI was 30.0 kg/m^2^ (IQR, 26.5–34.6). The weighted proportions of women and non‐Hispanic white participants were 46.89% and 63.42%, respectively. Compared with participants without MASLD, those with MASLD were more likely to be male and had higher ALT, AST, US FLI, and BMI values, together with lower ALM/BMI. The MASLD group also had higher weighted prevalence of dyslipidemia, hypertension, and sarcopenia and a lower proportion of physically active participants. Participants were categorized into quartiles according to their US FLI scores (Q1: 0.14–19.62, Q2: 19.63–37.39, Q3: 37.40–60.69, and Q4: 60.70–99.45) (Table S2).

**TABLE 1:** Characteristics of the study population by MASLD status (weighted) (NHANES 1999–2006, 2011–2018).

Variable	Total (*N* = 2093)	Non‐MASLD (*N* = 834)	MASLD (*N* = 1259)	*p* value
Age (years)	51.00 (43.00, 58.00)	51.00 (42.00, 58.00)	52.00 (43.00, 59.00)	0.05
ALT (U/L)	24.00 (18.00, 32.00)	20.00 (16.00, 26.00)	27.00 (21.00, 37.00)	< 0.01
AST (U/L)	23.00 (19.00, 28.00)	21.00 (18.00, 25.00)	24.00 (19.00, 29.00)	< 0.01
US FLI	36.57 (19.63, 59.86)	16.14 (9.55, 23.04)	54.20 (39.70, 71.50)	< 0.01
BMI (kg/m^2^)	30.00 (26.50, 34.60)	26.90 (24.10, 30.00)	32.30 (29.07, 37.40)	< 0.01
ALM/BMI	0.75 (0.61,0.91)	0.76 (0.62, 0.94)	0.75 (0.60, 0.88)	0.01
Female, *n* (%)	991 (46.89)	405 (50.88)	586 (44.15)	0.04
Race, *n* (%)				< 0.01
Mexican American	464 (9.59)	111 (6.83)	353 (11.49)	
Non‐Hispanic black	411 (11.17)	234 (15.72)	177 (8.05)	
Non‐Hispanic white	803 (63.42)	298 (59.11)	505 (66.39)	
Other	415 (15.81)	191 (18.35)	224 (14.07)	
PIR, *n* (%)				0.21
< 1	436 (13.96)	169 (13.74)	267 (14.10)	
1 ∼ 3	904 (38.74)	342 (36.34)	562 (40.39)	
> 3	753 (47.30)	323 (49.92)	430 (45.51)	
Educational level, *n* (%)				0.61
College or higher	957 (53.99)	418 (55.56)	539 (52.90)	
High school	441 (24.62)	179 (23.23)	262 (25.57)	
Less than high school	695 (21.40)	237 (21.20)	458 (21.53)	
Smoke, *n* (%)				0.02
Former	605 (28.56)	205 (24.28)	400 (31.50)	
Never	1141 (53.82)	483 (56.54)	658 (51.96)	
Now	347 (17.62)	146 (19.18)	201 (16.54)	
Physical activity, *n* (%)				0.04
No	877 (35.74)	315 (32.28)	562 (38.12)	
Yes	1216 (64.26)	519 (67.72)	697 (61.88)	
History of Hyperlipidemia, *n* (%)	1758 (84.44)	639 (76.96)	1119 (89.59)	< 0.01
History of Sarcopenia, *n* (%)	452 (17.10)	109 (10.23)	343 (21.83)	< 0.01
History of Hypertension, *n* (%)	1112 (49.70)	370 (38.89)	742 (57.14)	< 0.01

*Note:* MASLD: metabolic dysfunction–associated steatotic liver disease; AST: aspartate transferase; ALT: alanine transferase; ZJU: Zhejiang University Index.

Abbreviations: ALM, appendicular lean mass; BMI, body mass index; FSI, Framingham Steatosis Index; HSI, Hepatic Steatosis Index; PIR, poverty‐to‐income ratio; US FLI, US Fatty Liver Index.

### 3.2. The Association Between MASLD and Sarcopenia

After adjustment for potential confounders in Model 2, each 1‐SD increase in US FLI was associated with higher odds of sarcopenia (OR, 1.54; 95% CI, 1.32–1.79) (Table S3). When US FLI was analyzed as a categorical variable, participants in the third quartile (OR = 2.01, 95% CI: 1.21–3.35) and the fourth quartile (OR = 3.22, 95% CI: 2.02–5.13) had significantly higher odds of having sarcopenia than those in the lowest quartile. Univariable weighted logistic regression analysis showed a positive association between MASLD and sarcopenia among participants with T2DM or prediabetes (OR = 2.45; 95% CI: 1.85–3.25). After full adjustment for potential confounders, MASLD remained significantly associated with higher odds of sarcopenia (OR = 2.12; 95% CI: 1.56–2.86) (Table 2). Compared with the non‐MASLD subgroup, the MASLD subgroup had a significantly higher prevalence of sarcopenia (21.83% vs. 10.23%; *p* < 0.01) (Figure 2). There was no evidence of nonlinearity in the association between US FLI and sarcopenia in the study population (*p* for nonlinearity = 0.81) (Figure 3).

**TABLE 2:** The association between MASLD and sarcopenia (NHANES 1999–2006, 2011–2018).

Sarcopenia	Crude model OR (95% CI)	*p* value	Model 1 OR (95% CI)	*p* value	Model 2 OR (95% CI)	*p* value
MASLD						
No	Ref		Ref		Ref	
Yes	2.45 (1.85, 3.25)	< 0.01	2.22 (1.68, 2.95)	< 0.01	2.12 (1.56, 2.86)	< 0.01

*Note:* Crude model: no variables were adjusted. Model 1: adjusted for age, sex, and race. Model 2: additionally adjusted for educational levels, PIR, smoking status, physical activity, hypertension, and dyslipidemia. MASLD: metabolic dysfunction–associated steatotic liver disease.

Abbreviations: CI, confidence interval; OR, odds ratio; PIR, poverty‐to‐income ratio.

**FIGURE 2:**
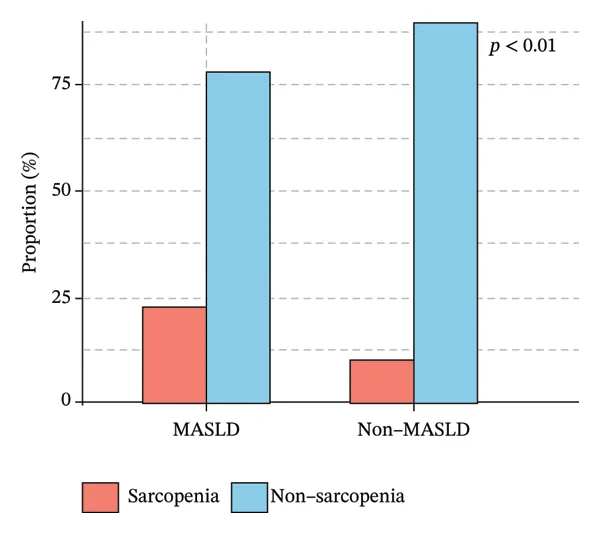
The prevalence of sarcopenia is closely associated with MASLD in US adults with T2DM or prediabetes (*p* < 0.01). MASLD: metabolic dysfunction–associated steatotic liver disease; T2DM: type 2 diabetes.

**FIGURE 3:**
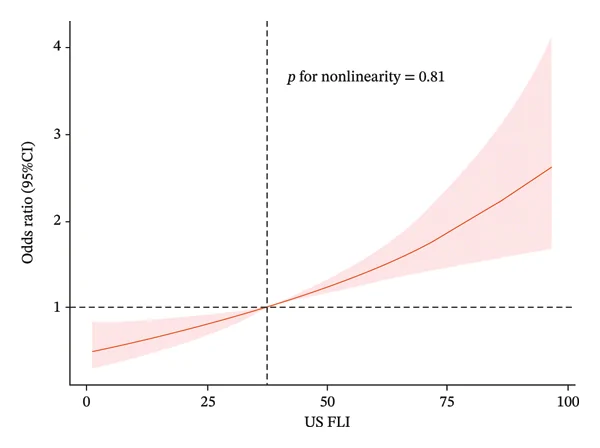
Restricted cubic spline analyses of the association between US FLI and the odds of sarcopenia. The model for the overall population was adjusted for age, sex, race, educational level, PIR, smoking status, physical activity, hypertension, and dyslipidemia. Solid lines represent estimated odds ratios, and shaded areas indicate 95% confidence intervals. US FLI, US Fatty Liver Index; PIR: poverty‐to‐income ratio.

### 3.3. Associations of MASLD With Inflammatory Markers and Their Associations With Sarcopenia

Table 3 displays the associations between MASLD and inflammatory markers derived from weighted multivariable linear regression analyses. After adjustment for potential confounders, MASLD was associated with higher levels of RAR (*β* = 0.09; 95% CI: 0.05–0.14; *p* < 0.01), WBC (*β* = 0.73; 95% CI: 0.50–0.95; *p* < 0.01), NPAR (*β* = 0.06; 95% CI: 0.03–0.09; *p* < 0.01), RDW (*β* = 0.15; 95% CI: 0.02–0.27; *p* = 0.02), and SIRI (*β* = 0.14; 95% CI: 0.03–0.25; *p* = 0.01). In contrast, MASLD was significantly associated with lower serum albumin levels (g/L) (*β* = −0.71; 95% CI: −1.13 to −0.29; *p* < 0.01). The associations between inflammatory biomarkers and sarcopenia are presented in Table 4. RAR, WBC, NPAR, RDW, and SIRI were positively associated with sarcopenia, whereas serum albumin showed an inverse association.

**TABLE 3:** The associations of MASLD and inflammatory biomarkers (NHANES 1999–2006, 2011–2018).

Inflammatory biomarkers	Crude model β (95% CI)	*p* value	Model 1 β (95% CI)	*p* value	Model 2 β (95% CI)	*p* value
RAR (rescaled)^a^	0.08 (0.03, 0.13)	< 0.01	0.11 (0.06, 0.16)	< 0.01	0.09 (0.05, 0.14)	< 0.01
WBC (× 10^9^/L)	0.83 (0.60, 1.06)	< 0.01	0.82 (0.58, 1.06)	< 0.01	0.73 (0.50, 0.95)	< 0.01
Albumin (g/L)	−0.71 (−1.11, −0.30)	< 0.01	−0.81 (−1.22, −0.40)	< 0.01	−0.71 (−1.13, −0.29)	< 0.01
NPAR	0.07 (0.04, 0.10)	< 0.01	0.06 (0.03, 0.09)	< 0.01	0.06 (0.03, 0.09)	< 0.01
RDW (%)	0.09 (−0.04, 0.23)	0.17	0.16 (0.02, 0.30)	0.02	0.15 (0.02, 0.27)	0.02
SIRI (× 10^9^/L)	0.18 (0.08, 0.29)	< 0.01	0.15 (0.04, 0.26)	0.01	0.14 (0.03, 0.25)	0.01

*Note:* Crude model: no variables were adjusted. Model 1: adjusted for age, sex, and race. Model 2: additionally adjusted for educational levels, PIR, smoking status, physical activity, hypertension, and dyslipidemia. The reported coefficients therefore refer to the rescaled RAR. NPAR was calculated as neutrophil percentage (%) divided by serum albumin concentration (g/L). RAR: red cell distribution width‐to‐albumin ratio; RDW: red cell distribution width; WBC: white blood cell count; MASLD: metabolic dysfunction–associated steatotic liver disease; *β*: regression coefficient.

Abbreviations: CI, confidence interval; NPAR, neutrophil percentage‐to‐albumin ratio; PIR, poverty‐to‐income ratio; SIRI, systemic inflammation response index.

^a^RAR was rescaled by dividing its original values by 0.1.

**TABLE 4:** The associations of inflammatory biomarkers and sarcopenia (NHANES 1999–2006, 2011–2018).

Inflammatory biomarkers	Crude model OR (95% CI)	*p* value	Model 1 OR (95% CI)	*p* value	Model 2 OR (95% CI)	*p* value
RAR (rescaled)^a^	1.73 (1.33,2.25)	< 0.01	2.10 (1.52,2.91)	< 0.01	1.94 (1.38,2.72)	< 0.01
WBC (× 10^9^/L)	1.10 (1.03,1.18)	0.01	1.12 (1.04,1.21)	< 0.01	1.10 (1.01,1.19)	0.02
Albumin (g/L)	0.93 (0.89,0.97)	< 0.01	0.92 (0.88,0.96)	< 0.01	0.93 (0.89,0.97)	< 0.01
NPAR	2.95 (1.61,5.43)	< 0.01	2.85 (1.47,5.50)	< 0.01	2.59 (1.33,5.05)	0.01
RDW (%)	1.15 (1.05,1.26)	< 0.01	1.24 (1.10,1.39)	< 0.01	1.21 (1.07,1.37)	< 0.01
SIRI (× 10^9^/L)	1.25 (1.10,1.42)	< 0.01	1.23 (1.06,1.41)	0.01	1.19 (1.04,1.37)	0.01

*Note:* Crude model: no variables were adjusted. Model 1: adjusted for age, sex, and race. Model 2: additionally adjusted for educational levels, PIR, smoking status, physical activity, hypertension, and dyslipidemia. The reported coefficients therefore refer to the rescaled RAR. NPAR was calculated as neutrophil percentage (%) divided by serum albumin concentration (g/L). RAR: red cell distribution width‐to‐albumin ratio; RDW: red cell distribution width; WBC: white blood cell count.

Abbreviations: CI, confidence interval; NPAR, neutrophil percentage‐to‐albumin ratio; OR, odds ratio; PIR, poverty‐to‐income ratio; SIRI, systemic inflammation response index.

^a^RAR was rescaled by dividing its original values by 0.1.

### 3.4. Potential Mediating Effects of Inflammatory Biomarkers

Statistical indirect associations involving RAR, WBC, serum albumin, NPAR, RDW, and SIRI were observed in separate mediation models of the association between MASLD and sarcopenia, with estimated mediation proportions of 10.35%, 9.14%, 8.98%, 6.47%, 4.28%, and 2.54%, respectively (Figure 4).

**FIGURE 4:**
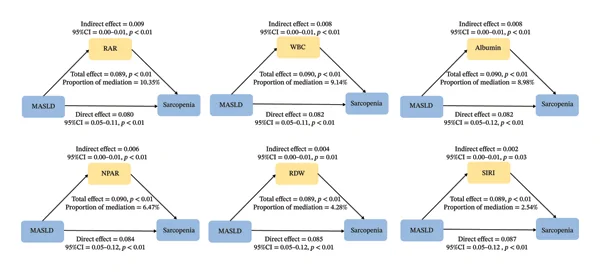
Mediation analyses of inflammatory biomarkers in the association between MASLD and sarcopenia. RAR: red cell distribution width‐to‐albumin ratio; RDW: red cell distribution width; WBC: white blood cell count; NPAR: neutrophil percentage‐to‐albumin ratio; SIRI: systemic inflammation response index; MASLD: metabolic dysfunction–associated steatotic liver disease.

### 3.5. Sensitivity Analyses Using Alternative Surrogate Indices of Hepatic Steatosis

After adjustment for the same potential confounders as in the primary analysis, the associations between MASLD and sarcopenia remained statistically significant across all alternative definitions. The adjusted ORs were 2.53 (95% CI: 1.73–3.70) using HSI, 2.26 (95% CI: 1.50–3.39) using ZJU, and 2.89 (95% CI: 1.99–4.18) using FSI, supporting the consistency of the association across these alternative surrogate definitions of MASLD (Table S4).

### 3.6. Subgroup Analyses

Subgroup analyses were performed to examine whether the association varied by age, sex, PIR, smoking status, physical activity, or hypertension status (Figure S1). No statistically significant interactions were detected for any of the examined subgroup variables (all *p* for interaction > 0.05).

## 4. Discussion

### 4.1. Major Findings

This cross‐sectional study included 2093 (weighted 9,501,956) US adults with T2DM or prediabetes. After adjustment for potential confounders, MASLD was significantly associated with higher odds of sarcopenia, and similar associations were observed when alternative hepatic steatosis indices were used to define MASLD. Separate mediation models suggested that several circulating inflammatory and nutritional biomarkers might be involved in this association, with estimated mediation proportions ranging from 2.54% to 10.35%. No significant interactions were detected in subgroup analyses.

### 4.2. Comparison With Other Studies

Sarcopenia is increasingly recognized as a progressive, generalized skeletal muscle disorder associated with an increased risk of falls, fractures, physical disability, and mortality [38]. In this study, MASLD was significantly associated with higher odds of sarcopenia among adults with T2DM or prediabetes. Although differences in study populations, designs, and disease definitions limit direct comparisons, this finding is broadly consistent with previous evidence linking hepatic steatosis to sarcopenia [39]. A 10‐year retrospective cohort study of 4398 adults found that lower muscle mass was associated with a higher risk of incident NAFLD, with a more pronounced association among nonobese individuals [40]. Using NHANES 2017–2018 data, Feng et al. reported an association between sarcopenia and MAFLD and suggested a potential mediating role of C‐reactive protein [41]. The present study extends this evidence by combining data from multiple survey cycles, focusing on adults with T2DM or prediabetes, and examining the potential mediating roles of several circulating inflammatory biomarkers.

Regarding inflammatory markers, Guo et al. reported associations between complete blood count–derived inflammatory indices and sarcopenia [42]. Another cross‐sectional study of 10,092 participants found that elevated platelet and WBC counts were independently associated with sarcopenia [43]. These findings provide a rationale for examining circulating inflammatory markers in the association between liver and muscle health. Our mediation analyses further suggested that these biomarkers might be involved in the association between MASLD and sarcopenia.

Beyond blood cell–derived inflammatory indices, our mediation analyses also suggested a potential involvement of serum albumin in the association between MASLD and sarcopenia. Previous studies have highlighted the clinical relevance of albumin in liver disease. For example, Kawanaka et al. found that lower albumin levels were associated with poorer outcomes in patients with biopsy‐confirmed NAFLD [44]. Although that study examined prognosis rather than sarcopenia, its findings suggest that albumin may reflect the overall health status of patients with liver disease. Albumin levels are influenced not only by inflammation but also by nutritional status, hepatic synthetic function, and protein loss [45, 46]. Therefore, the estimated mediation involving albumin in our study may reflect the combined influence of multiple physiological and pathological processes rather than a single inflammatory pathway. Taken together, these findings extend the evidence linking MASLD and sarcopenia to adults with T2DM or prediabetes and suggest that circulating inflammatory and nutritional biomarkers may help characterize this association.

### 4.3. Potential Mechanisms

Several shared biological processes may help explain the association between MASLD and sarcopenia. Insulin resistance and metabolic dysfunction may provide a shared pathophysiological basis for hepatic steatosis and impaired muscle health. Reduced insulin‐stimulated glucose utilization and glycogen synthesis in skeletal muscle may redirect dietary carbohydrates toward hepatic de novo lipogenesis, favoring hepatic lipid accumulation [47]. In parallel, resistance to the anabolic effects of insulin may blunt muscle protein synthesis, potentially compromising the maintenance of muscle mass [48]. Together, these disturbances in glucose, lipid, and protein metabolism offer a plausible biological explanation for the coexistence of MASLD and sarcopenia. In addition to insulin resistance, inflammatory processes may provide another biological link between MASLD and sarcopenia. Steatotic hepatocytes and intrahepatic immune cells can release inflammatory signals that promote neutrophil recruitment and the accumulation of monocyte‐derived macrophages [49–51]. Under persistent inflammatory conditions, excessive neutrophil activation and the release of proteases, oxidants, cytokines, and neutrophil extracellular traps may further exacerbate tissue injury and metabolic disturbances [52–54]. Meanwhile, lipid accumulation in skeletal muscle and adipose tissue may enhance proinflammatory signaling, while impaired mitochondrial β‐oxidation may increase reactive oxygen species production and lipid peroxidation, disrupting muscle metabolic homeostasis [55, 56]. Inflammatory mediators, including IL‐6 and TNF‐α, may also alter the balance between muscle protein synthesis and degradation, compromising muscle mass maintenance [8, 9]. These interconnected processes provide a plausible biological context for interpreting our biomarker findings.

Within this framework, WBC, NPAR, and SIRI may provide indirect information about systemic immune‐inflammatory status, whereas RDW, albumin, and their composite measure, RAR, may additionally reflect physiological and pathological processes beyond inflammation. RDW is influenced by erythropoiesis and nutritional status, while albumin is associated with inflammation, nutritional status, and hepatic synthetic function. Accordingly, the estimated mediation effects may reflect the combined contributions of multiple processes rather than a single inflammatory pathway. Furthermore, RAR and NPAR share albumin as a denominator, and RAR also incorporates RDW, resulting in overlapping information across these biomarkers. Their estimated mediation proportions should therefore be interpreted with these interrelationships in mind, without treating the findings for individual biomarkers as evidence of distinct biological pathways or inferring the relative importance of specific mechanisms solely from the magnitude of these proportions.

### 4.4. Strengths and Limitations

The strengths of this study include the use of a nationally representative sample, which supports the generalizability of the findings to U.S. adults with T2DM or prediabetes. In addition, this study extends previous research by examining the potential mediating roles of multiple inflammatory biomarkers in the association between MASLD and sarcopenia within this population. Sensitivity analyses using alternative hepatic steatosis indices (HSI, ZJU, and FSI) yielded consistent results, further supporting the robustness of our findings. Collectively, these findings highlight the potential importance of sarcopenia screening among individuals with MASLD, particularly those with T2DM or prediabetes. This study has several limitations. First, because of its cross‐sectional design, this study could not establish temporal or causal relationships among MASLD, chronic inflammation, and sarcopenia. Therefore, the mediation findings should be interpreted as statistical associations rather than causal evidence. Second, MASLD was operationally defined using the US FLI, an indirect surrogate index rather than an imaging‐ or biopsy‐based assessment of hepatic steatosis. Although US FLI has been validated in large epidemiological studies, its use may have introduced some degree of misclassification bias. Finally, sarcopenia was defined solely using DXA‐derived ALM/BMI, without assessing muscle strength or physical performance. This mass‐based definition may not fully capture clinical sarcopenia and could have introduced outcome misclassification, potentially affecting the observed associations. Some potentially relevant factors, such as dietary intake, medication use, and other unmeasured clinical characteristics, were not included in the analyses and may have influenced the observed associations.

## 5. Conclusion

In conclusion, MASLD was significantly associated with higher odds of sarcopenia among individuals with T2DM or prediabetes. Exploratory mediation analyses identified statistical indirect associations involving inflammatory biomarkers. These findings highlight the importance of considering sarcopenia alongside MASLD in this population. Lifestyle interventions, including increased physical activity, may have a role in comprehensive management; however, prospective and interventional studies are needed to clarify temporal relationships, investigate potential mechanisms, and evaluate their benefits in individuals with these coexisting conditions.

## Author Contributions

Lu Long: methodology, software, formal analysis, data curation, and writing–original draft. Zhiwen Shu: methodology, investigation, data curation, and validation. Weixue Wang: methodology, investigation, and data curation. Lei He: methodology and investigation. Yuwen Jiang: methodology and data curation. Qing Li: methodology and investigation. Xiaozhong Li: methodology and investigation. Juxiang Li: conceptualization, methodology, writing–review and editing, supervision, and project administration.

## Funding

This research did not receive any specific grant from funding agencies in the public, commercial, or not‐for‐profit sectors.

## Ethics Statement

The research protocol received clearance from the NHANES Institutional Review Board and was conducted in adherence to the Declaration of Helsinki, with all NHANES individuals submitting signed informed consent.

## Consent

Please see the Ethics Statement.

## Conflicts of Interest

The authors declare no conflicts of interest.

## Supporting Information

Additional supporting information can be found online in the Supporting Information section.

## Supporting information


**Supporting Information** The supporting information includes Tables S1–S4 and Figure S1, which provide additional details regarding the study methodology, baseline characteristics, and supporting analyses, supporting the robustness and comprehensiveness of the study findings.

## Data Availability

The datasets generated and researched in this investigation were available from the NHANES database, https://www.cdc.gov/nchs/nhanes/.

## References

[1] Cruz-Jentoft A. J. , Baeyens J. P. , Bauer J. M. et al., Sarcopenia: European Consensus on Definition and Diagnosis: Report of the European Working Group on Sarcopenia in Older People, Age and Ageing. (2010) 39, 412–423. 10.1093/ageing/afq034 PMC2886201 20392703

[2] Morley J. E. , Sarcopenia: Diagnosis and Treatment, The Journal of Nutrition, Health & Aging. (2008) 12, no. 7, 452–456, 10.1007/bf02982705. PMC12892578 18615226

[3] Wild S. H. , Lazarus J. V. , Spearman C. W. et al., MASLD Prevalence, Incidence and Global Aspects, Diabetologia. (2026) . 10.1007/s00125-026-06726-1 PMC13627207 42168648

[4] Wei X. , Liu X. , Zhao J. , Zhang Y. , Qiu L. , and Zhang J. , Association of Sarcopenia and Physical Activity on the Severity of Metabolic Dysfunction-Associated Steatotic Liver Disease Among United States Adults: NHANES 2017–2018, Frontiers in aging. (2025) 6, 10.3389/fragi.2025.1573170. PMC12106442 40433168

[5] Zhang F. , Liu L. , and Li W. , Correlation of Sarcopenia With Progression of Liver Fibrosis in Patients With Metabolic Dysfunction-Associated Steatotic Liver Disease: A Study From Two Cohorts in China and the United States, Nutrition Journal. (2025) 24, no. 1, 10.1186/s12937-025-01081-0. PMC11730808 39810142

[6] Huby T. and Gautier E. L. , Immune Cell-Mediated Features of Non-Alcoholic Steatohepatitis, Nature Reviews Immunology. (2022) 22, no. 7, 429–443, 10.1038/s41577-021-00639-3. PMC8570243 34741169

[7] Kim Y. , Park Y. , Rho H. , Yao T. , Gao B. , and Hwang S. , Inflammation in MASLD Progression and Cancer, JHEP Reports: Innovation in Hepatology. (2025) 7, no. 8, 10.1016/j.jhepr.2025.101414. PMC12266516 40671831

[8] Beyer I. , Mets T. , and Bautmans I. , Chronic Low-Grade Inflammation and Age-Related Sarcopenia, Current Opinion in Clinical Nutrition and Metabolic Care. (2012) 15, no. 1, 12–22, 10.1097/mco.0b013e32834dd297. 22108098

[9] Han J. W. , Kim D. I. , Nam H. C. , Chang U. I. , Yang J. M. , and Song D. S. , Association Between Serum Tumor Necrosis Factor-α and Sarcopenia in Liver Cirrhosis, Clinical and Molecular Hepatology. (2022) 28, no. 2, 219–231, 10.3350/cmh.2021.0082. PMC9013623 34281295

[10] Lu Z. , Hu Y. , He H. et al., Associations of Muscle Mass, Strength, and Quality With Diabetes and the Mediating Role of Inflammation in Two National Surveys From China and the United States, Diabetes Research and Clinical Practice. (2024) 214, 10.1016/j.diabres.2024.111783. 39002932

[11] Wang G. , Zhao Y. , Li Z. et al., Association Between Novel Inflammatory Markers and Non-Alcoholic Fatty Liver Disease: A Cross-Sectional study, European Journal of Gastroenterology and Hepatology. (2024) 36, no. 2, 203–209, 10.1097/meg.0000000000002686. PMC10906204 38047735

[12] Hong H. C. , Hwang S. Y. , Choi H. Y. et al., Relationship Between Sarcopenia and Nonalcoholic Fatty Liver Disease: The Korean Sarcopenic Obesity Study, Hepatology. (2014) 59, no. 5, Baltimore, Md, 1772–1778, 10.1002/hep.26716. 23996808

[13] Sun H. , Saeedi P. , Karuranga S. et al., IDF Diabetes Atlas: Global, Regional and Country-Level Diabetes Prevalence Estimates for 2021 and Projections for 2045, Diabetes Research and Clinical Practice. (2022) 183, 10.1016/j.diabres.2021.109119. PMC11057359 34879977

[14] Stumvoll M. , Goldstein B. J. , and van Haeften T. W. , Type 2 Diabetes: Principles of Pathogenesis and Therapy, Lancet (London, England). (2005) 365, no. 9467, 1333–1346, 10.1016/s0140-6736(05)61032-x. 15823385

[15] Młynarska E. , Czarnik W. , Dzieża N. et al., Type 2 Diabetes Mellitus: New Pathogenetic Mechanisms, Treatment and the Most Important Complications, International Journal of Molecular Sciences. (2025) 26, no. 3, 10.3390/ijms26031094. PMC11817707 39940862

[16] Takamura T. , Misu H. , Ota T. , and Kaneko S. , Fatty Liver as a Consequence and Cause of Insulin Resistance: Lessons From Type 2 Diabetic Liver, Endocrine Journal. (2012) 59, no. 9, 745–763, 10.1507/endocrj.ej12-0228. 22893453

[17] Hong S. H. and Choi K. M. , Sarcopenic Obesity, Insulin Resistance, and Their Implications in Cardiovascular and Metabolic Consequences, International Journal of Molecular Sciences. (2020) 21, no. 2, 10.3390/ijms21020494. PMC7013734 31941015

[18] Liccini A. and Malmstrom T. K. , Frailty and Sarcopenia as Predictors of Adverse Health Outcomes in Persons With Diabetes Mellitus, Journal of the American Medical Directors Association. (2016) 17, no. 9, 846–851, 10.1016/j.jamda.2016.07.007. 27569712

[19] Targher G. , Corey K. E. , Byrne C. D. , and Roden M. , The Complex Link Between NAFLD and Type 2 Diabetes Mellitus–Mechanisms and Treatments, Nature Reviews Gastroenterology & Hepatology. (2021) 18, no. 9, 599–612, 10.1038/s41575-021-00448-y. 33972770

[20] Zou X. , Zhou X. , Zhu Z. , and Ji L. , Novel Subgroups of Patients With Adult-Onset Diabetes in Chinese and US Populations, Lancet Diabetes and Endocrinology. (2019) 7, no. 1, 9–11, 10.1016/s2213-8587(18)30316-4. 30577891

[21] Ruhl C. E. and Everhart J. E. , Fatty Liver Indices in the Multiethnic United States National Health and Nutrition Examination Survey, Alimentary Pharmacology and Therapeutics. (2015) 41, no. 1, 65–76, 10.1111/apt.13012. 25376360

[22] EASL-EASD-EASO Clinical Practice Guidelines on the Management of Metabolic Dysfunction-Associated Steatotic Liver Disease (MASLD), Journal of Hepatology. (2024) 81, 492–542. 10.1016/j.jhep.2024.04.031 38851997

[23] Studenski S. A. , Peters K. W. , Alley D. E. et al., The FNIH Sarcopenia Project: Rationale, Study Description, Conference Recommendations, and Final Estimates, Journals of Gerontology. Series A, Biological Sciences and Medical Sciences. (2014) 69, no. 5, 547–558, 10.1093/gerona/glu010. PMC3991146 24737557

[24] Tan M. Y. , Zhang Y. J. , Zhu S. X. , Wu S. , Zhang P. , and Gao M. , The Prognostic Significance of Stress Hyperglycemia Ratio in Evaluating All-Cause and Cardiovascular Mortality Risk Among Individuals Across Stages 0-3 of Cardiovascular-Kidney-Metabolic Syndrome: Evidence From Two Cohort Studies, Cardiovascular Diabetology. (2025) 24, no. 1, 10.1186/s12933-025-02689-6. PMC11934678 40128747

[25] Kurkiewicz K. , Gąsior M. , and Szyguła-Jurkiewicz B. E. , Markers of Malnutrition, Inflammation, and Tissue Remodeling are Associated With 1-Year Outcomes in Patients With Advanced Heart Failure, Polish Archives of Internal Medicine. (2023) . 10.20452/pamw.16411 36633195

[26] Yu B. , Li M. , Yu Z. et al., Red Blood Cell Distribution Width to Albumin Ratio (RAR) is Associated With Low Cognitive Performance in American Older Adults: NHANES 2011-2014, BMC Geriatrics. (2025) 25, no. 1, 10.1186/s12877-025-05800-4. PMC11887108 40055657

[27] Zhang Y. , He Z. , Li K. et al., Neutrophil-to-Albumin Ratio: A Novel Predictor of Osteoporosis in Rheumatoid Arthritis, Frontiers in Immunology. (2025) 16, 10.3389/fimmu.2025.1666884. PMC12484169 41041308

[28] Chen Y. , Lian W. , Wu L. et al., Joint Association of Estimated Glucose Disposal Rate and Systemic Inflammation Response Index With Mortality in Cardiovascular-Kidney-Metabolic Syndrome Stage 0-3: A Nationwide Prospective Cohort Study, Cardiovascular Diabetology. (2025) 24, no. 1, 10.1186/s12933-025-02692-x. PMC11955130 40158167

[29] Pan J. , Zhou Y. , Pang N. , and Yang L. , Dietary Niacin Intake and Mortality Among Individuals With Nonalcoholic Fatty Liver Disease, JAMA Network Open. (2024) 7, no. 2, 10.1001/jamanetworkopen.2023.54277. PMC10835509 38300619

[30] Lin J. , Zhuo X. , Bardenheier B. et al., Cost-Effectiveness of the 2014 U.S. Preventive Services Task Force (USPSTF) Recommendations for Intensive Behavioral Counseling Interventions for Adults With Cardiovascular Risk Factors, Diabetes Care. (2017) 40, no. 5, 640–646, 10.2337/dc16-1186. 28213373

[31] Whelton P. K. , Carey R. M. , Aronow W. S. et al., ACC/AHA/AAPA/ABC/ACPM/AGS/APhA/ASH/ASPC/NMA/PCNA Guideline for the Prevention, Detection, Evaluation, and Management of High Blood Pressure in Adults: A Report of the American College of Cardiology/American Heart Association Task Force on Clinical Practice Guidelines, Journal of the American College of Cardiology. (2018) 71, e127–e248. 10.1016/j.jacc.2017.11.006 29146535

[32] Lee J. H. , Kim D. , Kim H. J. et al., Hepatic Steatosis Index: A Simple Screening Tool Reflecting Nonalcoholic Fatty Liver Disease, Digestive and Liver Disease. Official Journal of the Italian Society of Gastroenterology and the Italian Association for the Study of the Liver. (2010) 42, no. 7, 503–508, 10.1016/j.dld.2009.08.002. 19766548

[33] Wang J. , Xu C. , Xun Y. , Lu Z. , Shi J. , and Yu C. , ZJU Index: A Novel Model for Predicting Nonalcoholic Fatty Liver Disease in a Chinese Population, Scientific Reports. (2015) 5, no. 1, 10.1038/srep16494. PMC4645098 26568423

[34] Long M. T. , Pedley A. , Colantonio L. D. et al., Development and Validation of the Framingham Steatosis Index to Identify Persons With Hepatic Steatosis, Clinical Gastroenterology and Hepatology: The Official Clinical Practice Journal of the American Gastroenterological Association. (2016) 14, no. 8, 1172–1180.e1172, 10.1016/j.cgh.2016.03.034. PMC4955680 27046482

[35] Zou H. , Ma X. , Zhang F. , and Xie Y. , Comparison of the Diagnostic Performance of Twelve Noninvasive Scores of Metabolic Dysfunction-Associated Fatty Liver Disease, Lipids in Health and Disease. (2023) 22, no. 1, 10.1186/s12944-023-01902-3. PMC10481547 37674196

[36] Petermann-Rocha F. , Gray S. R. , Forrest E. et al., Associations of Muscle Mass and Grip Strength With Severe NAFLD: A Prospective Study of 333,295 UK Biobank Participants, Journal of Hepatology. (2022) 76, no. 5, 1021–1029, 10.1016/j.jhep.2022.01.010. 35085594

[37] Gan M. , Chen B. , Qin M. , Zhang L. , and Wang X. , Association Between Atherogenic Index of Plasma (AIP) and All-Cause and Cardiovascular Mortality in Patients With Metabolic Dysfunction-Associated Steatotic Liver Disease (MASLD): A Cohort Study Based on NHANES 1999-2018, Cardiovascular Diabetology. (2025) 24, no. 1, 10.1186/s12933-025-02941-z. PMC12512302 41073997

[38] Cruz-Jentoft A. J. , Bahat G. , Bauer J. et al., Sarcopenia: Revised European Consensus on Definition and Diagnosis, Age and Ageing. (2019) 48, no. 4, 16–31, 10.1093/ageing/afz046. PMC6322506 30312372

[39] Malik A. , Javaid S. , Malik M. I. , and Qureshi S. , Relationship Between Sarcopenia and Metabolic Dysfunction-Associated Steatotic Liver Disease (MASLD): A Systematic Review and Meta-Analysis, Annals of Hepatology. (2024) 29, no. 6, 10.1016/j.aohep.2024.101544. 39214253

[40] Lee M. J. , Kim E. H. , Bae S. J. et al., Age-Related Decrease in Skeletal Muscle Mass is an Independent Risk Factor for Incident Nonalcoholic Fatty Liver Disease: A 10-Year Retrospective Cohort Study, Gut and Liver. (2019) 13, no. 1, 67–76, 10.5009/gnl18070. PMC6347003 30037166

[41] Feng Z. , Zhao F. , Wang Z. , Tang X. , Xie Y. , and Qiu L. , The Relationship Between Sarcopenia and Metabolic Dysfunction-Associated Fatty Liver Disease Among the Young and Middle-Aged Populations, BMC Gastroenterology. (2024) 24, no. 1, 10.1186/s12876-024-03192-0. PMC10943823 38491346

[42] Guo B. , Liu X. , Si Q. et al., Associations of CBC-Derived Inflammatory Indicators With Sarcopenia and Mortality in Adults: Evidence From Nhanes 1999 ∼ 2006, BMC Geriatrics. (2024) 24, no. 1, 10.1186/s12877-024-05012-2. PMC11100216 38755603

[43] Lee H. S. , Koh I. H. , Kim H. S. , and Kwon Y. J. , Platelet and White Blood Cell Count are Independently Associated With Sarcopenia: A Nationwide Population-Based Study, Thrombosis Research. (2019) 183, 36–44, 10.1016/j.thromres.2019.09.007. 31614293

[44] Kawanaka M. , Nishino K. , Ishii K. et al., Combination of Type IV Collagen 7S, Albumin Concentrations, and Platelet Count Predicts Prognosis of Non-Alcoholic Fatty Liver Disease, World Journal of Hepatology. (2021) 13, no. 5, 571–583, 10.4254/wjh.v13.i5.571. PMC8173338 34131471

[45] Amouzandeh M. , Sundström A. , Wahlin S. , Wernerman J. , Rooyackers O. , and Norberg Å , Albumin and Fibrinogen Synthesis Rates in Advanced Chronic Liver Disease, American Journal of Physiology–Gastrointestinal and Liver Physiology. (2023) 325, no. 5, G391–g397, 10.1152/ajpgi.00072.2023. 37605837

[46] Evans D. C. , Corkins M. R. , Malone A. et al., The Use of Visceral Proteins as Nutrition Markers: An ASPEN Position Paper, Nutrition in Clinical Practice: Official Publication of the American Society for Parenteral and Enteral Nutrition. (2021) 36, no. 1, 22–28, 10.1002/ncp.10588. 33125793

[47] Flannery C. , Dufour S. , Rabøl R. , Shulman G. I. , and Petersen K. F. , Skeletal Muscle Insulin Resistance Promotes Increased Hepatic de Novo Lipogenesis, Hyperlipidemia, and Hepatic Steatosis in the Elderly, Diabetes. (2012) 61, no. 11, 2711–2717, 10.2337/db12-0206. PMC3478531 22829450

[48] Rasmussen B. B. , Fujita S. , Wolfe R. R. et al., Insulin Resistance of Muscle Protein Metabolism in Aging, FASEB Journal: Official Publication of the Federation of American Societies for Experimental Biology. (2006) 20, no. 6, 768–769, 10.1096/fj.05-4607fje. PMC2804965 16464955

[49] Gadd V. L. , Skoien R. , Powell E. E. et al., The Portal Inflammatory Infiltrate and Ductular Reaction in Human Nonalcoholic Fatty Liver Disease, Hepatology. (2014) 59, no. 4, Baltimore, Md, 1393–1405, 10.1002/hep.26937. 24254368

[50] Rensen S. S. , Slaats Y. , Nijhuis J. et al., Increased Hepatic Myeloperoxidase Activity in Obese Subjects With Nonalcoholic Steatohepatitis, American Journal of Pathology. (2009) 175, no. 4, 1473–1482, 10.2353/ajpath.2009.080999. PMC2751544 19729473

[51] Guillot A. and Tacke F. , Liver Macrophages: Old Dogmas and New Insights, Hepatology Communications. (2019) 3, no. 6, 730–743, 10.1002/hep4.1356. PMC6545867 31168508

[52] Jorch S. K. and Kubes P. , An Emerging Role for Neutrophil Extracellular Traps in Noninfectious Disease, Nature Medicine. (2017) 23, no. 3, 279–287, 10.1038/nm.4294. 28267716

[53] Soehnlein O. , Steffens S. , Hidalgo A. , and Weber C. , Neutrophils as Protagonists and Targets in Chronic Inflammation, Nature Reviews Immunology. (2017) 17, no. 4, 248–261, 10.1038/nri.2017.10. 28287106

[54] Peiseler M. and Kubes P. , More Friend Than Foe: The Emerging Role of Neutrophils in Tissue Repair, Journal of Clinical Investigation. (2019) 129, no. 7, 2629–2639, 10.1172/jci124616. PMC6597202 31205028

[55] Legaki A. I. , Moustakas I. I. , Sikorska M. , Papadopoulos G. , Velliou R. I. , and Chatzigeorgiou A. , Hepatocyte Mitochondrial Dynamics and Bioenergetics in Obesity-Related Non-Alcoholic Fatty Liver Disease, Current Obesity Reports. (2022) 11, no. 3, 126–143, 10.1007/s13679-022-00473-1. PMC9399061 35501558

[56] Romanello V. and Sandri M. , Mitochondrial Quality Control and Muscle Mass Maintenance, Frontiers in Physiology. (2015) 6, 10.3389/fphys.2015.00422. PMC4709858 26793123

