# Supplementary material for: Mediation Analysis of Inflammatory Biomarkers in the Association Between Metabolic Dysfunction–Associated Steatotic Liver Disease and Sarcopenia Among US Adults With Type 2 Diabetes or Prediabetes
Source: Int J Endocrinol. 2026 Nov 15;2026:3157645. doi: 10.1155/ije/3157645 (PMC13770318; doi:10.1155/ije/3157645)
Supplement: Supplementary file 1 [file IJE-2026-3157645-s001.docx]

**Mediation analysis of inflammatory biomarkers in the association between metabolic dysfunction-associated steatotic liver disease and sarcopenia among US adults with type 2 diabetes or prediabetes.**

Lu Long^1^^#^, M.D., Zhiwen Shu^2#^, M.D., Weixue Wang^1^, M.D., Lei He^1^, M.D., Yuwen Jiang^1^, M.D., Qing Li^1^, M.D., Ph.D., Xiaozhong Li^1^*, M.D., Ph.D, Juxiang Li^1^*, M.D., Ph.D.

^1^ Department of Cardiology, the Second Affiliated Hospital, Jiangxi Medical College, Nanchang University, Nanchang, Jiangxi, China

^2^ Department of Orthopedics, the Second Affiliated Hospital, Jiangxi Medical College, Nanchang University, Nanchang, Jiangxi, China

^#^Co-first author: Lu Long and Zhiwen Shu.

*Correspondence:

Xiaozhong Li

lxz1919197@163.com

Juxiang Li

[ljx912@126.com](mailto:ljx912@126.com)

**Table of Contents**

| Table S1 | Calculation formulas for the HSI, ZJU, and FSI. | P3 |
| --- | --- | --- |
| Table S2 | Baseline characteristics according to quartiles of US FLI (NHANES 1999–2006, 2011–2018). | P4 |
| Table S3 | The association between US FLI and sarcopenia (NHANES 1999–2006, 2011–2018). | P6 |
| Table S4 | Sensitivity analysis of the association between MASLD and sarcopenia using alternative hepatic steatosis indices (HSI, ZJU, and FSI) (NHANES 1999–2006, 2011–2018) | P7 |
| Figure S1 | Forest plots of relationships between MASLD and sarcopenia with subgroups (NHANES 1999–2006, 2011–2018). | P9 |

**Table S1. Calculation formulas for the HSI, ZJU, and FSI.**

|  | Formulas |
| --- | --- |
| HSI | 8 × [ALT (U/L) / AST (U/L)] + BMI (kg/m^2^) (+ 2, if diabetes; + 2, if female) |
| ZJU | BMI (kg/m^2^) + FPG (mmol/L) + TG (mmol/L) + 3 × [ALT (U/L) / AST (U/L)] (+ 2, if female) |
| FSI | -7.981 + 0.011 × Age (years) - 0.146 × Sex (female = 1, male = 0) + 0.173 × BMI (kg/m^2^) + 0.007 × TG (mg/dL) + 0.593 × Hypertension (yes = 1, no = 0) + 0.789 × Diabetes (yes = 1, no = 0) + 1.1 × [ALT (U/L) / AST (U/L) ≥ 1.33 (yes = 1, no = 0)] |

**Table S2. Baseline characteristics according to quartiles of US FLI** **(NHANES 1999–2006, 2011–2018).**

| Variable | Q1 (n = 523) | Q2 (n = 523) | Q3 (n = 523) | Q4 (n = 524) | P-value |
| --- | --- | --- | --- | --- | --- |
| Age (years) | 50.00 (40.00,57.00) | 53.00 (44.00,59.00) | 52.00 (44.00,60.00) | 51.00 (42.00,59.00) | 0.003 |
| ALT (U/L) | 19.00 (15.00,24.00) | 23.00 (19.00,31.00) | 26.00 (19.00,33.00) | 31.00 (22.00,46.00) | < 0.01 |
| AST (U/L) | 21.00 (18.00,25.00) | 22.00 (19.00,26.00) | 23.00 (19.00,28.00) | 25.00 (20.00,32.00) | < 0.01 |
| US FLI | 11.26 (7.03,15.30) | 28.26 (23.79,32.78) | 47.20 (41.75,53.41) | 75.63 (67.18,85.54) | < 0.01 |
| BMI | 26.00 (23.27,28.80) | 29.00 (26.50,32.10) | 31.11 (28.50,35.28) | 36.30 (31.66,40.94) | < 0.01 |
| ALM/BMI | 0.77 (0.63,0.97) | 0.73 (0.59,0.92) | 0.74 (0.60,0.90) | 0.75 (0.61,0.87) | 0.01 |
| Female, n (%) | 249 (51.40) | 259 (49.16) | 250 (45.55) | 233 (41.14) | 0.09 |
| Race, n (%) |  |  |  |  | < 0.01 |
| Mexican American | 57 (5.83) | 107 (9.00) | 126 (11.00) | 174 (12.66) |  |
| Non-Hispanic black | 168 (18.59) | 102 (10.61) | 89 (10.09) | 52 (5.31) |  |
| non-Hispanic white | 181 (58.13) | 201 (64.08) | 201 (62.26) | 220 (69.26) |  |
| other | 117 (17.45) | 113 (16.31) | 107 (16.65) | 78 (12.77) |  |
| PIR, n (%) |  |  |  |  | 0.03 |
| <1 | 108 (13.98) | 109 (12.66) | 115 (15.68) | 104 (13.64) |  |
| 1~3 | 203 (34.03) | 227 (39.92) | 214 (35.26) | 260 (45.69) |  |
| >3 | 212 (51.99) | 187 (47.42) | 194 (49.06) | 160 (40.67) |  |
| Educational level, n (%) |  |  |  |  | 0.7 |
| college or higher | 280 (58.11) | 233(52.10) | 238(54.06) | 206(51.77) |  |
| high school | 112 (23.49) | 111(25.38) | 100(23.43) | 118(26.10) |  |
| less than high school | 131 (18.40) | 179(22.52) | 185(22.51) | 200(22.12) |  |
| Smoking, n (%) |  |  |  |  | 0.02 |
| never | 300 (57.28) | 306 (57.65) | 269 (47.99) | 266 (51.91) |  |
| former | 120 (22.88) | 139 (25.21) | 174 (34.44) | 172 (32.16) |  |
| now | 103 (19.84) | 78 (17.13) | 80 (17.57) | 86 (15.93) |  |
| Physical activity, n (%) |  |  |  |  | 0.01 |
| no | 190 (31.04) | 207 (31.28) | 220 (38.05) | 260 (43.11) |  |
| yes | 333 (68.96) | 316 (68.72) | 303 (61.95) | 264 (56.89) |  |
| History of Hyperlipidemia, n (%) | 377 (73.51) | 437 (83.13) | 468 (90.04) | 476 (91.46) | < 0.01 |
| History of Sarcopenia, n (%) | 50 (8.37) | 99 (13.69) | 128 (19.30) | 175 (27.55) | < 0.01 |
| History of Hypertension, n (%) | 216 (35.48) | 271 (47.37) | 302 (56.74) | 323 (59.77) | < 0.01 |

US FLI:US Fatty Liver Index; ALM: appendicular lean mass; BMI: body mass index; PIR: poverty-to-income ratio; AST: Aspartate transferase; ALT: alanine transferase;

**Table S3.** **The association between US FLI and sarcopenia (NHANES 1999–2006, 2011–2018).**

|  | Case/total | Crude model  OR (95%) | P-value | Model 1  OR (95%) | P-value | Model 2  OR (95%) | P-value |
| --- | --- | --- | --- | --- | --- | --- | --- |
| Per 1SD increase |  | 1.65 (1.43,1.90) | <0.01 | 1.60 (1.38,1.85) | <0.01 | 1.54 (1.32,1.79) | <0.01 |
| Quartiles |  |  |  |  |  |  |  |
| Q1 (0.14~19.62) | 50/523 | Ref |  | Ref |  | Ref |  |
| Q2 (19.63~37.392) | 99/523 | 1.74 (1.12,2.69) | 0.01 | 1.44 (0.92,2.25) | 0.11 | 1.39 (0.88,2.21) | 0.16 |
| Q3 (37.394~60.69) | 128/523 | 2.62 (1.64,4.18) | <0.01 | 2.11 (1.32,3.38) | <0.01 | 2.01 (1.21,3.35) | 0.01 |
| Q4 (60.70~99.45) | 175/524 | 4.16 (2.67,6.50) | <0.01 | 3.56 (2.30,5.51) | <0.01 | 3.22 (2.02,5.13) | <0.01 |
| P for trend |  |  | <0.01 |  | <0.01 |  | <0.01 |

Crude model: no variables were adjusted

Model 1: adjusted for age, sex and race

Model 2 additionally adjusted for educational levels, PIR, smoking status, physical activity, hypertension and dyslipidemia.

US FLI: US Fatty Liver Index; PIR: poverty-to-income ratio;

**Table S4. Sensitivity analysis of the association between MASLD and sarcopenia using alternative hepatic steatosis indices (HSI, ZJU, and FSI) (NHANES 1999–2006, 2011–2018)**

| **Sarcopenia** | Crude model  OR (95%CI) | P-value | Model 1  OR (95%CI) | P-value | Model 2  OR (95%CI) | P-value |
| --- | --- | --- | --- | --- | --- | --- |
| **MASLD (HSI)** | | | | | | |
| No | Ref |  | Ref |  | Ref |  |
| Yes | 2.13 (1.51,3.01) | < 0.01 | 2.64 (1.82,3.83) | < 0.01 | 2.53 (1.73,3.70) | < 0.01 |
| **MASLD (ZJU)** |  |  |  |  |  |  |
| No | Ref |  | Ref |  | Ref |  |
| Yes | 2.19 (1.49,3.20) | < 0.01 | 2.44 (1.66,3.59) | < 0.01 | 2.26 (1.50,3.39) | < 0.01 |
| **MASLD (FSI)** |  |  |  |  |  |  |
| No | Ref |  | Ref |  | Ref |  |
| Yes | 2.72 (1.99,3.71) | < 0.01 | 2.91 (2.10,4.04) | < 0.01 | 2.89 (1.99,4.18) | < 0.01 |

Crude model: no variables were adjusted

Model 1: adjusted for age, sex and race

Model 2 additionally adjusted for educational levels, PIR, smoking status, physical activity, hypertension and dyslipidemia.

MASLD: metabolic dysfunction-associated steatotic liver disease; PIR: poverty-to-income ratio; ZJU: Zhejiang University index; FSI: Framingham steatosis index; HSI: Hepatic steatosis index; OR：odds ratio; CI：confidence interval;

**Figure S1. Forest plots of relationships between MASLD and sarcopenia with subgroups (NHANES 1999–2006, 2011–2018)**

MASLD: metabolic dysfunction-associated steatotic liver disease; PIR: poverty-to-income ratio;
